# Vorasidenib and ivosidenib in IDH1-mutant low-grade glioma: a randomized, perioperative phase 1 trial

**DOI:** 10.1038/s41591-022-02141-2

**Published:** 2023-02-23

**Authors:** Ingo K. Mellinghoff, Min Lu, Patrick Y. Wen, Jennie W. Taylor, Elizabeth A. Maher, Isabel Arrillaga-Romany, Katherine B. Peters, Benjamin M. Ellingson, Marc K. Rosenblum, Saewon Chun, Kha Le, Ania Tassinari, Sung Choe, Youssef Toubouti, Steven Schoenfeld, Shuchi S. Pandya, Islam Hassan, Lori Steelman, Jennifer L. Clarke, Timothy F. Cloughesy

**Affiliations:** 1 Memorial Sloan Kettering Cancer Center, New York, NY USA; 2 Agios Pharmaceuticals, Cambridge, MA USA; 3 Dana-Farber Cancer Institute, Boston, MA USA; 4 University of California San Francisco, San Francisco, CA USA; 5 University of Texas Southwestern Medical Center, Dallas, TX USA; 6 Massachusetts General Hospital, Harvard Medical School, Boston, MA USA; 7 Duke University Medical Center, Durham, NC USA; 8 University of California, Los Angeles, Los Angeles, CA USA; 9 California University of Science and Medicine, Colton, CA USA; 10 Aligos Therapeutics, South San Francisco, CA USA; 11 Servier Pharmaceuticals LLC, Boston, MA USA; 12 Present Address: Mersana Therapeutics, Cambridge, MA USA; 13 Present Address: Sage Therapeutics, Cambridge, MA USA

**Keywords:** CNS cancer, Drug development

## Abstract

Vorasidenib and ivosidenib inhibit mutant forms of isocitrate dehydrogenase (mIDH) and have shown preliminary clinical activity against m*IDH* glioma. We evaluated both agents in a perioperative phase 1 trial to explore the mechanism of action in recurrent low-grade glioma (IGG) and select a molecule for phase 3 testing. Primary end-point was concentration of d-2-hydroxyglutarate (2-HG), the metabolic product of mIDH enzymes, measured in tumor tissue from 49 patients with m*IDH1*-R132H nonenhancing gliomas following randomized treatment with vorasidenib (50 mg or 10 mg once daily, q.d.), ivosidenib (500 mg q.d. or 250 mg twice daily) or no treatment before surgery. Tumor 2-HG concentrations were reduced by 92.6% (95% credible interval (CrI), 76.1–97.6) and 91.1% (95% CrI, 72.0–97.0) in patients treated with vorasidenib 50 mg q.d. and ivosidenib 500 mg q.d., respectively. Both agents were well tolerated and follow-up is ongoing. In exploratory analyses, 2-HG reduction was associated with increased DNA 5-hydroxymethylcytosine, reversal of ‘proneural’ and ‘stemness’ gene expression signatures, decreased tumor cell proliferation and immune cell activation. Vorasidenib, which showed brain penetrance and more consistent 2-HG suppression than ivosidenib, was advanced to phase 3 testing in patients with m*IDH* LGGs. Funded by Agios Pharmaceuticals, Inc. and Servier Pharmaceuticals LLC; ClinicalTrials.gov number NCT03343197.

## Abstract (web summary)

The dual IDH1/IDH2 inhibitor vorasidenib exhibited better brain permeability and target engagement than ivosidenib in a pilot perioperative randomized clinical trial in patients with IDH1-mutant glioma.

## Main

Gliomas are a heterogeneous group of primary brain tumors that are associated with diffuse brain infiltration and premature death^1,2^. Central nervous system (CNS) World Health Organization (WHO) grade 2 and 3 gliomas initially grow at a slower rate than glioblastomas (CNS WHO grade 4) but later transform into aggressive tumors with neovascularization and contrast enhancement on magnetic resonance imaging (MRI)^1,3–5^. Diffuse gliomas in adults cannot be cured by surgery, radiation or chemotherapy and are associated with considerable disease- and treatment-associated morbidity^1,6^. There is an unmet need for new therapeutic options with favorable safety profiles and the potential for longer treatment duration^1,6^.

Most LGGs in adults harbor mutations in the genes encoding the metabolic enzyme IDH1 or, rarely, IDH2 (refs. ^1,7^). Cancer-associated mutations confer the enzyme with the neomorphic ability to catalyze the production of 2-HG^8,9^. 2-HG accumulates in tumor tissue and inhibits 2-oxoglutarate-dependent enzymes, a family of enzymes that includes the TET family of 5-methylcytosine (5mC) hydroxylases, the JmjC family of histone demethylases and many other enzymes controlling a wide range of cellular functions^10^. Compared with gliomas without *IDH* mutations, m*IDH* gliomas follow a distinct molecular pathogenesis, with a characteristic pattern of genomic and epigenetic alterations^11–14^.

Small-molecule inhibitors of mIDH enzymes have emerged as a new strategy for the treatment of m*IDH* cancers. Ivosidenib, an inhibitor of the mIDH1 enzyme, is approved for the treatment of subsets of m*IDH1* acute myeloid leukemias and previously treated, locally advanced/metastatic cholangiocarcinomas, and has shown preliminary antitumor activity in patients with m*IDH1* glioma and chondrosarcoma^15–17^. Vorasidenib, a dual inhibitor of mIDH1 and mIDH2 enzymes, was designed for improved penetrance of the blood–brain barrier and has also shown preliminary antitumor activity in patients with m*IDH* glioma^18,19^. Before advancing ivosidenib or vorasidenib to randomized phase 3 evaluation, we conducted the current perioperative study to document inhibition of the mIDH enzyme and mIDH pathway-related pharmacodynamic (PD) effects in on-treatment tumor biopsies in a side-by-side evaluation of both agents. We examined two different dosing schedules for each agent to identify the optimal biological dose in patients with m*IDH1* glioma.

## Results

### Patient characteristics

**Fig. 1:**
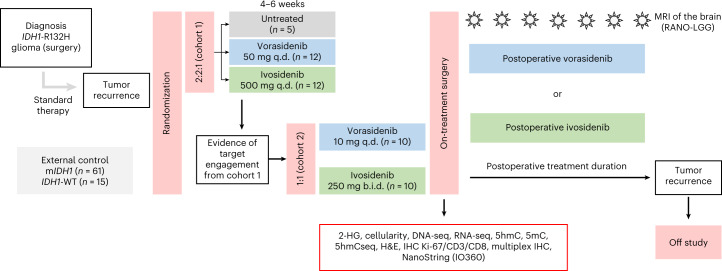
Study design. An overview of the study design is shown. All patients could opt to receive the study drug postoperatively. After surgery, patients in the untreated control group were re-randomized 1:1 to either vorasidenib 50 mg q.d. or ivosidenib 500 mg q.d. Based on the PD and pharmacokinetic results of cohort 1, alternative dose regimens of vorasidenib and/or ivosidenib were to be tested in cohort 2.

**Extended Data Fig. 1:**
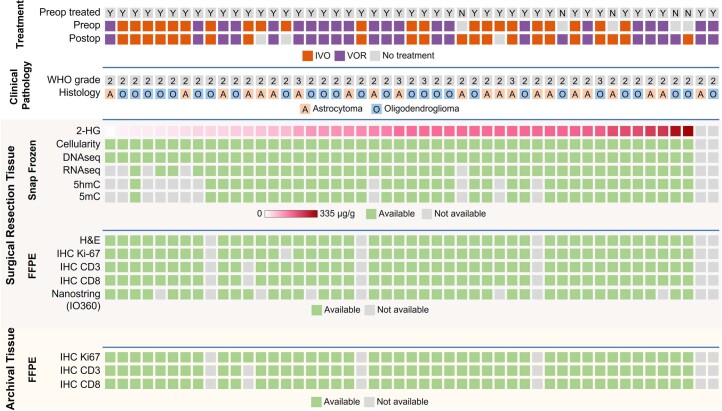
Overview of biospecimen analysis. 2-HG denotes D-2-hydroxyglutarate, 5hmC 5-hydroxymethylcytosine, 5mC 5-methylcytosine, DNAseq DNA sequencing, FFPE formalin-fixed paraffin embedded, H&E hematoxylin and eosin, IHC, immunohistochemistry; IVO, ivosidenib; Postop, postoperative; Preop, preoperative; RNAseq, RNA sequencing; VOR, vorasidenib; WHO, World Health Organization.

Patients were assessed for eligibility between March 2018 and April 2019 across seven sites in the USA. Enrollment was completed in April 2019 and follow-up of the study remains ongoing. As of 29 April 2020 (analysis cutoff date), 49 patients overall were randomized before surgery. Patients in cohort 1 were randomized in a 2:2:1 ratio to ivosidenib 500 mg q.d., vorasidenib 50 mg q.d. or no treatment before surgery. After documenting inhibition of the m*IDH1* enzyme in cohort 1, cohort 2 was opened to test alternative dose regimens and patients were randomized 1:1 to ivosidenib 250 mg twice daily (b.i.d.) or vorasidenib 10 mg q.d. Treated patients received 28 (+7) d of drug up to and including the day of surgery. All patients had the option to receive postoperative treatment until disease progression or unacceptable toxicity (Fig. 1). Intra-patient dose escalation was permitted per protocol. Tumor and blood samples were collected and analyzed per protocol. On-treatment tumor tissue was compared with tumor tissue from a previous surgery whenever possible (Extended Data Fig. 1).

**Extended Data Fig. 2:**
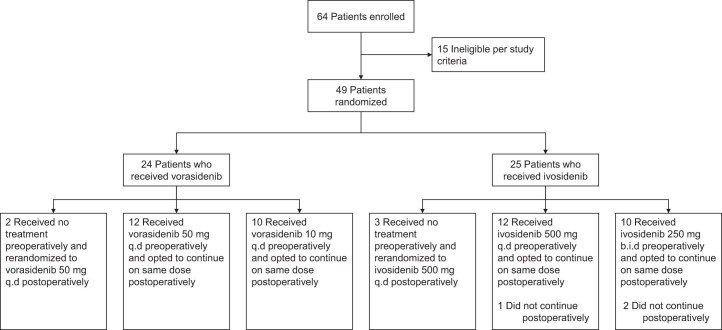
Trial profile. B.i.d. denotes twice daily, and q.d. once daily.

Overall, 24 patients received at least one dose of vorasidenib and 25 patients received at least one dose of ivosidenib. Patients who had been randomized to the control arm before surgery were re-randomized after surgery to either vorasidenib 50 mg q.d. (*n* = 2) or ivosidenib 500 mg q.d. (*n* = 3) (Extended Data Fig. 2). At the time of the analysis cutoff date, 17 (70.8%) patients remained on vorasidenib treatment. Five (20.8%) patients discontinued vorasidenib owing to disease progression and two (8.3%) discontinued per investigator decision; 15 (60.0%) patients remained on ivosidenib treatment, three (12.0%) did not continue ivosidenib postoperatively, six (24.0%) discontinued ivosidenib owing to disease progression and one (4.0%) discontinued owing to an adverse event (AE).

Demographic and baseline characteristics were similar for the vorasidenib and ivosidenib cohorts (Table 1). Most patients had WHO grade 2 tumors (43 of 49; 87.8%) based on the most recent pathology before screening. All patients had at least one previous surgery; 24 (49.0%) received previous systemic therapy and 14 (28.6%) received previous radiation therapy.

**Table 1:** Demographic and baseline characteristics

	Vorasidenib			Ivosidenib		
	50 mg q.d. (*n* = 14)^a^	10 mg q.d. (*n* = 10)	Total (*n* = 24)	500 mg q.d. (*n* = 15)^b^	250 mg b.i.d. (*n* = 10)	Total (*n* = 25)
Median (range) age (years)	48.5 (31–61)	49.5 (34–75)	49 (31–75)	37 (24–57)	40.5 (19–66)	37 (19–66)
Male/female, *n* (%)	10 (71.4)/4 (28.6)	6 (60.0)/4 (40.0)	16 (66.7)/8 (33.3)	10 (66.7)/5 (33.3)	7 (70.0)/3 (30.0)	17 (68.0)/8 (32.0)
KPS score at baseline, *n* (%)						
100%	4 (28.6)	4 (40.0)	8 (33.3)	7 (46.7)	4 (40.0)	11 (44.0)
90%	8 (57.1)	5 (50.0)	13 (54.2)	7 (46.7)	5 (50.0)	12 (48.0)
80%	2 (14.3)	1 (10.0)	3 (12.5)	1 (6.7)	–	1 (4.0)
Missing	–	–	–	–	1 (10.0)	1 (4.0)
WHO tumor grade at screening, *n* (%)						
Grade 2	13 (92.9)	9 (90.0)	22 (91.7)	13 (86.7)	8 (80.0)	21 (84.0)
Grade 3	1 (7.1)	1 (10.0)	2 (8.3)	2 (13.3)	2 (20.0)	4 (16.0)
Histological subtype, *n* (%)						
Oligodendroglioma	8 (57.1)	5 (50.0)	13 (54.2)	8 (53.3)	4 (40.0)	12 (48.0)
Astrocytoma	6 (42.9)	5 (50.0)	11 (45.8)	6 (40.0)	5 (50.0)	11 (44.0)
Anaplastic oligodendroglioma	–	–	–	1 (6.7)	–	1 (4.0)
Anaplastic oligoastrocytoma	–	–	–	–	1 (10.0)	1 (4.0)
1p19q status (if known), *n* (%)						
Intact^c^	5 (35.7)	5 (50.0)	10 (41.7)	5 (33.3)	4 (40.0)	9 (36.0)
Codeleted	8 (57.1)	4 (40.0)	12 (50.0)	8 (53.3)	5 (50.0)	13 (52.0)
Not determined	1 (7.1)	1 (10.0)	2 (8.3)	2 (13.3)	1 (10.0)	3 (12.0)
Previous surgery, *n* (%)	14 (100)	10 (100)	24 (100)	15 (100)	10 (100)	25 (100)
Previous radiation therapy, *n* (%)	4 (28.6)	3 (30.0)	7 (29.2)	5 (33.3)	2 (20.0)	7 (28.0)
Previous systemic therapy, *n* (%)	6 (42.9)	4 (40.0)	10 (41.7)	9 (60.0)	5 (50.0)	14 (56.0)

KPS, Karnofsky performance status.

^a^Includes two patients who were assigned to the control arm before surgery.

^b^Includes three patients who were assigned to the control arm before surgery.

^c^Includes patients with no 1p19q codeletion, 1p deletion only or 19q deletion only.

### Tumor 2-HG and drug concentrations

Tumors from 40 of 49 patients, including all five untreated patients, were included in the tissue analyses. Nine patients were excluded from the tissue analysis because they did not have enough remaining tissue (*n* = 2), m*IDH1* was not confirmed in resected tissue (*n* = 3) or they received incorrect drug doses before surgery (*n* = 4).

**Fig. 2:**
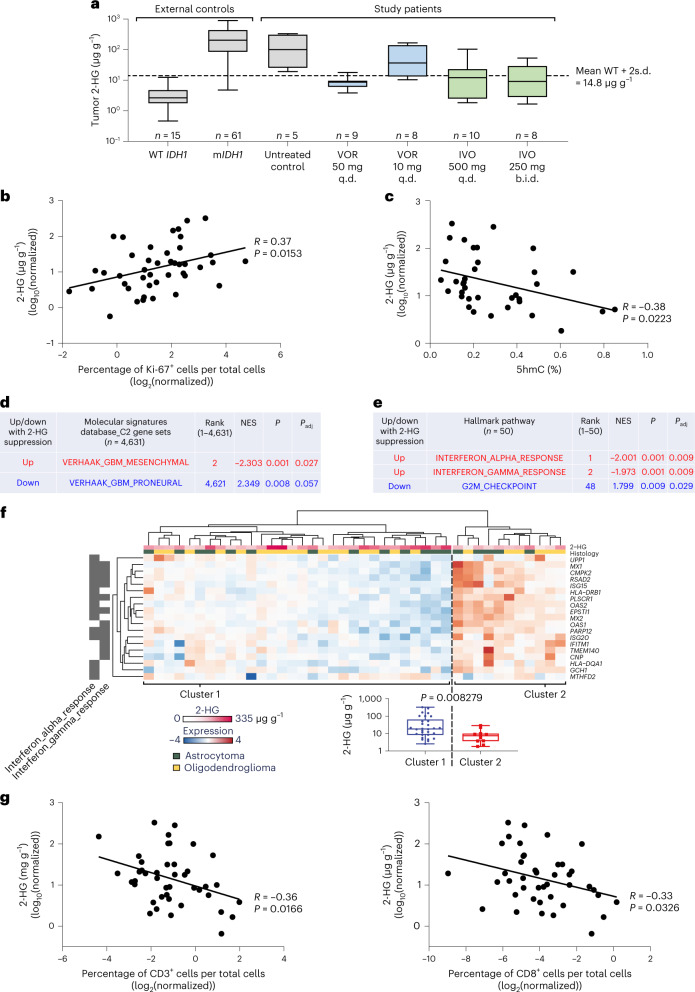
Tumor 2-HG concentration and associated molecular changes. **a**, 2-HG concentrations in external control and evaluable on-study resected tumor samples. Horizontal lines denote median values; boxes denote the 25th to 75th percentiles and the whiskers go from the smallest to the largest values. Colors indicate dose cohorts represented in Fig. 1. **b**, The percentage of Ki-67^+^ cells from on-treatment tumor samples in association with 2-HG. **c**, Levels of DNA hydroxymethylation from on-treatment tumor samples in association with 2-HG. The percentage of 5hmC was calculated as the ratio 5hmC:cytosine. **d**,**e**, Modulation of selected molecular pathways on 2-HG suppression. *P* values are adjusted for multiplicity as described in Methods. **f**, An unsupervised clustering of 2-HG–associated genes in the IFN-α/γ response pathways. Box plot: the horizontal lines denote median values, the boxes denote 25th to 75th percentiles and the whiskers go from the smallest to the largest values (*n* = 30 for cluster 1 and *n* = 11 for cluster 2; two-sided *P* value generated with Student’s *t*-test). **g**, The densities of CD3^+^ and CD8^+^ T cells from on-treatment tumor samples in association with 2-HG. For **b**, **c** and **g**, two-sided *P* values are simple linear regressions. IVO, ivosidenib; VOR, vorasidenib.

In the external control group of archival tumors, the mean (s.d.) tumor 2-HG concentration was 3.7 (3.1) µg g^−1^ in wild-type (WT) *IDH* gliomas and 276.8 (231.4) µg g^−1^ in m*IDH1* gliomas. The mean (s.d.) tumor 2-HG concentration in patients who did not receive study treatment before surgery (untreated controls) was 154.9 (146.9) µg g^−1^. Mean (s.d.) tumor 2-HG concentrations in patients who received treatment before surgery were 8.9 (4.1) µg g^−1^ (vorasidenib 50 mg q.d.), 67.5 (65.4) µg g^−1^ (vorasidenib 10 mg q.d.), 20.9 (30.7) µg g^−1^ (ivosidenib 500 mg q.d.) and 16.8 (18.1) µg g^−1^ (ivosidenib 250 mg b.i.d.) (Fig. 2a).

**Extended Data Fig. 3:**
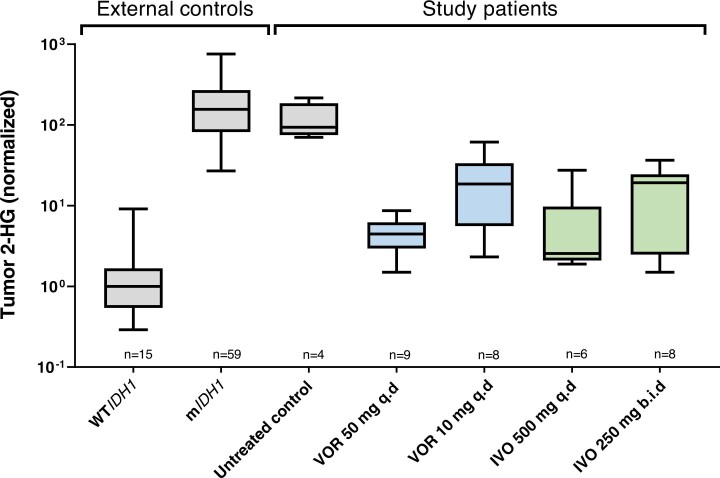
Normalized tumor 2-HG. Concentrations of 2-HG in resected tumor tissue were normalized for tumor cellularity as determined by hematoxylin and eosin staining, where available. Normalized 2-HG was calculated as 2-HG divided by the density of m*IDH* cells, where density of m*IDH* cells was calculated as the cellularity (number of total cells/mm^2^) × *IDH* VAF × 2. Samples with unavailable VAF data were excluded. Horizontal lines denote median values; boxes denote 25th to 75th percentiles; whiskers go from the smallest to the largest values. 2-HG denotes D-2-hydroxyglutarate; b.i.d., twice daily; *IDH*, isocitrate dehydrogenase; IVO, ivosidenib; m*IDH*, mutant *isocitrate dehydrogenase*; q.d., once daily; VAF, variant allele frequency; VOR, vorasidenib; WT, wild type.

The posterior mean percentage reduction in tumor 2-HG relative to the combined data from all untreated control tumors was 92.6% (95% CrI, 76.1–97.6) with vorasidenib 50 mg q.d. and 91.1% (95% CrI, 72.0–97.0) with ivosidenib 500 mg q.d., respectively. Correction of tumor 2-HG concentrations for tumor cellularity (‘normalized 2-HG’) reduced the variability in each treatment cohort (Extended Data Fig. 3).

Tumor concentrations of vorasidenib and ivosidenib were well above the reported half-maximal inhibitory concentration (IC_50_) for inhibition of the m*IDH1*-R132H allele for vorasidenib and ivosidenib, respectively^18,20^. Tumor:plasma ratios were considerably higher for vorasidenib than for ivosidenib (Supplementary Table 1).

### Safety

All patients proceeded to surgery as planned without any treatment-related delays. Both drugs were well tolerated. AEs were similar to previous studies of these agents and are listed in Table 2. Treatment-emergent AEs are listed in Supplementary Table 2.

**Table 2:** Adverse events

Event, *n* (%)	All grades^a^			Grade 3 and higher^a^		
Vorasidenib	Overall (*n* = 24)	10 mg q.d. (*n* = 10)	50 mg q.d. (*n* = 14)	Overall (*n* = 24)	10 mg q.d. (*n* = 10)	50 mg q.d. (*n* = 14)
Patients with ≥1 AE, *n (*%)	24 (100)	10 (100)	14 (100)	7 (29.2)	2 (20.0)	5 (35.7)
Most common AEs among vorasidenib-treated patients, *n* (%)^a^						
Nausea	10 (41.7)	5 (50.0)	5 (35.7)	0	0	0
Headache	10 (41.7)	5 (50.0)	5 (35.7)	0	0	0
Diarrhea	7 (29.2)	2 (20.0)	5 (35.7)	0	0	0
Fatigue	7 (29.2)	3 (30.0)	4 (28.6)	0	0	0
Alanine aminotransferase increased	5 (20.8)	0	5 (35.7)	1 (4.2)	0	1 (7.1)
Constipation	5 (20.8)	2 (20.0)	3 (21.4)	0	0	0
Insomnia	5 (20.8)	3 (30.0)	2 (14.3)	0	0	0
Aspartate aminotransferase increased	4 (16.7)	1 (10.0)	3 (21.4)	0	0	0
Anemia	4 (16.7)	2 (20.0)	2 (14.3)	1 (4.2)	1 (10.0)	0
Abdominal pain	4 (16.7)	1 (10.0)	3 (21.4)	0	0	0
Memory impairment	4 (16.7)	0	4 (28.6)	0	0	0
Tinnitus	3 (12.5)	1 (10.0)	2 (14.3)	0	0	0
Dyspepsia	3 (12.5)	1 (10.0)	2 (14.3)	0	0	0
Upper respiratory tract infection	3 (12.5)	2 (20.0)	1 (7.1)	0	0	0
Weight decreased	3 (12.5)	1 (10.0)	2 (14.3)	0	0	0
Hyperglycemia	3 (12.5)	1 (10.0)	2 (14.3)	1 (4.2)	0	1 (7.1)
Hypocalcemia	3 (12.5)	2 (20.0)	1 (7.1)	0	0	0
Hypophosphatemia	3 (12.5)	1 (10.0)	2 (14.3)	1 (4.2)	0	1 (7.1)
Aura	3 (12.5)	0	3 (21.4)	0	0	0
**Ivosidenib**	**Overall (*n*** **=** **25)**	**250** **mg b.i.d. (*n*** **=** **10)**	**500** **mg q.d. (*n*** **=** **15)**	**Overall (*n*** **=** **25)**	**250** **mg b.i.d. (*n*** **=** **10)**	**500** **mg q.d. (*n*** **=** **15)**
Patients with ≥1 AE, *n* (%)	25 (100)	10 (100)	15 (100)	6 (24.0)	1 (10.0)	5 (33.3)
Most common AEs among ivosidenib-treated patients^a^, *n* (%)						
Headache	9 (36.0)	4 (40.0)	5 (33.3)	0	0	0
Anemia	9 (36.0)	2 (20.0)	7 (46.7)	0	0	0
Diarrhea	7 (28.0)	2 (20.0)	5 (33.3)	0	0	0
Seizure	7 (28.0)	4 (40.0)	3 (20.0)	0	0	0
Hypocalcemia	7 (28.0)	1 (10.0)	6 (40.0)	0	0	0
Cough	6 (24.0)	1 (10.0)	5 (33.3)	0	0	0
Nasal congestion	6 (24.0)	2 (20.0)	4 (26.7)	0	0	0
Hypokalemia	6 (24.0)	2 (20.0)	4 (26.7)	0	0	0
Nausea	6 (24.0)	2 (20.0)	4 (26.7)	0	0	0
Hyperglycemia	5 (20.0)	1 (10.0)	4 (26.7)	0	0	0
Insomnia	5 (20.0)	2 (20.0)	3 (20.0)	0	0	0
Upper respiratory tract infection	4 (16.0)	1 (10.0)	3 (20.0)	0	0	0
Constipation	4 (16.0)	1 (10.0)	3 (20.0)	0	0	0
White blood cell count decreased	4 (16.0)	0	4 (26.7)	0	0	0
Anxiety	4 (16.0)	1 (10.0)	3 (20.0)	0	0	0
Pruritus	4 (16.0)	0	4 (26.7)	0	0	0
Fatigue	3 (12.0)	0	3 (20.0)	0	0	0
Aspartate aminotransferase increased	3 (12.0)	1 (10.0)	2 (13.3)	0	0	0
Electrocardiogram Q–T prolonged	3 (12.0)	0	3 (20.0)	0	0	0
Lymphocyte count decreased	3 (12.0)	0	3 (20.0)	0	0	0
Decreased appetite	3 (12.0)	1 (10.0)	2 (13.3)	0	0	0
Hyponatremia	3 (12.0)	1 (10.0)	2 (13.3)	1 (4.0)	0	1 (6.7)
Paresthesia	3 (12.0)	1 (10.0)	2 (13.3)	0	0	0
Depression	3 (12.0)	1 (10.0)	2 (13.3)	0	0	0

The safety population included all patients who received at least one dose of the study treatment pre- or postoperatively, categorized by assigned (that is, randomized) dose.

^a^All grade AEs reported in ≥10% of patients in the vorasidenib or ivosidenib arms and their corresponding grade 3 and higher frequencies are shown. Other reported grade 3 and higher AEs among vorasidenib-treated patients were brain abscess, tooth infection, aphasia, brain edema and hydrocephalus (each *n* = 1; 4.2%). Other reported grade 3 and higher AEs among ivosidenib-treated patients were leukopenia, subdural hematoma, invasive ductal breast carcinoma, brain edema, brain injury, hemiparesis, syncope, mental status changes and pneumothorax (each *n* = 1; 4.0%).

### IDH pathway-related molecular and cellular changes

**Extended Data Fig. 4:**
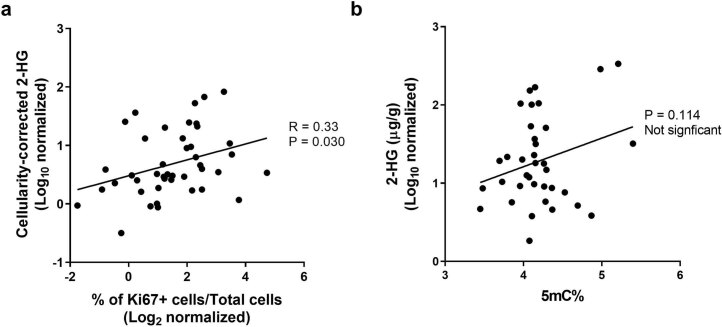
Correlation between 2-HG tumor concentrations and Ki-67 and DNA 5mC content. **a**,**b**, Simple linear regression of the percentage of Ki-67+ cells from on-treatment tumor samples in association with cellularity-corrected 2-HG (**a**) or percentage of 5mC content of DNA in association with tumor 2-HG (**b**). Cellularity-corrected 2-HG = 2-HG/cellularity.

Staining of resected tumor tissue with an antibody against the Ki-67 antigen, a marker for tumor cell proliferation, showed a positive correlation between tumor cell proliferation and tumor 2-HG concentrations (Fig. 2b), even after tumor 2-HG concentrations were corrected for cellularity (Extended Data Fig. 4a).

We observed an inverse correlation between tumor 2-HG concentrations and DNA 5-hydroxymethylcytosine (5hmC) content (Fig. 2c). There was no correlation between tumor 2-HG and DNA 5-methylcytosine (5mC) content (Extended Data Fig. 4b).

**Extended Data Fig. 5:**
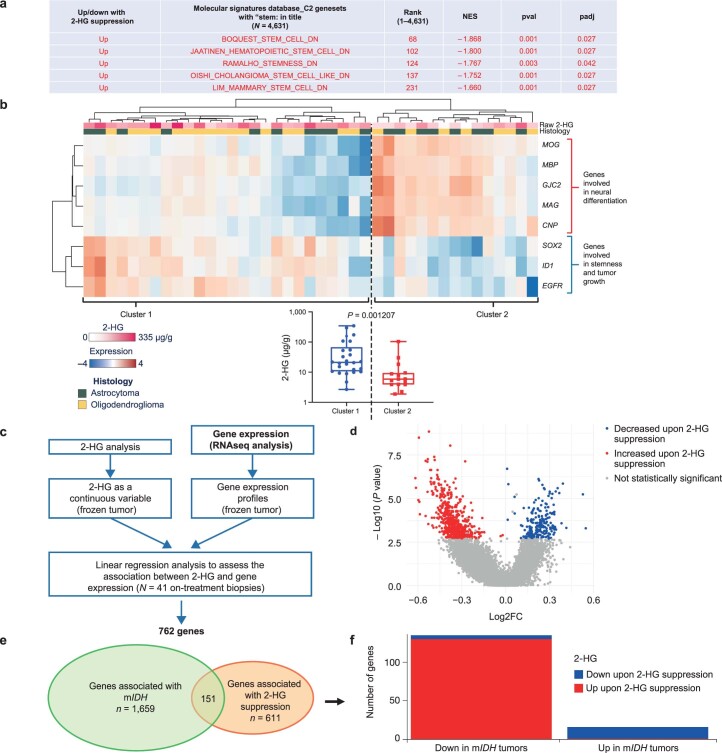
Gene expression analysis of frozen tissue samples. **a**, Modulation of top stemness-related pathways associated with cellular differentiation in the central nervous system upon 2-HG suppression. ‘DN’ indicates that genes in these pathways are downregulated in stem cells. Padj is P value adjusted for multiple comparisons as described in the Methods: if padj<0.05, the pathway is significantly associated with 2-HG suppression. **b**, Unsupervised clustering of 2-HG–associated genes involved in neuronal differentiation and stemness (N = 41). Box plot: horizontal lines denote median values, boxes denote 25th to 75th percentiles, whiskers go from the smallest to the largest values; two-sided *P* value generated with Student’s t test. **c**, Methodology for the determination of genes associated with 2-HG suppression. **d**, Volcano plot highlighting genes associated with 2-HG suppression. **e**, Venn diagram between genes associated with *IDH* mutations derived by comparing wild-type *IDH* and m*IDH* low-grade glioma in The Cancer Genome Atlas dataset and genes associated with 2-HG suppression as shown in Extended Data Fig. 5a. **f**, Plot of the 151 overlapping genes derived from Panel **a**. Note that the majority of genes that are downregulated in m*IDH* tumors (left bar) are upregulated upon 2-HG suppression and vice versa (right bar). 2-HG denotes D-2-hydroxyglutarate; FC, fold change; *IDH1, isocitrate dehydrogenase*; m*IDH1*, mutant *isocitrate dehydrogenase*; NES, normalized enrichment score; padj, adjusted P value pval, P value; RNAseq, RNA sequencing.

Genome-wide RNA expression profiling and data analysis, using established molecular pathway annotations (Molecular Signatures Database, C2 Pathways)^21^, showed that low tumor 2-HG was associated with a reversal of the ‘proneural’ gene expression signature (Fig. 2d and Supplementary Table 3), a molecular hallmark of m*IDH* gliomas^22^ and downregulation of genes linked to stem cell properties in a variety of cancers (Extended Data Fig. 5a,b). The last finding is consistent with the reported effect of 2-HG on cellular differentiation^23–27^.

Among gene sets that represent specific biological states or processes (Molecular Signatures Database, Hallmark Pathways), the interferon (IFN)-α and IFN-γ pathways were the most highly upregulated pathways at low tumor 2-HG concentrations. In contrast, genes associated with cell-cycle progression (G2M_Checkpoint) were suppressed at low tumor 2-HG levels (Fig. 2e and Supplementary Table 4).

Using linear regression analysis, we identified 762 genes that were induced or repressed in response to 2-HG suppression (Extended Data Fig. 5c,d). This list included genes associated with *IDH* mutations in both astrocytomas and oligodendrogliomas (Extended Data Fig. 5e,f), genes associated with cellular differentiation in the CNS (Extended Data Fig. 5a,b) and gene sets related to immune cell activation (Fig. 2f).

**Extended Data Fig. 6:**
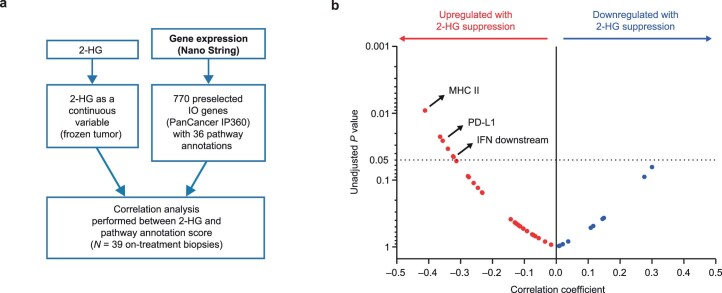
Examination of formalin-fixed paraffin-embedded tumor tissue from surgical samples and association with 2-HG suppression. **a**, The methodology for the determination of the Nanostring IO360 pathways associated with 2-HG suppression. **b**, Volcano plot highlighting selected pathways (gene expression analyzed using nSolver 4.0 and normalized to control genes, *P* values unadjusted) associated with 2-HG suppression. 2-HG denotes D-2-hydroxyglutarate, IFN interferon, MHC II major histocompatibility complex class II, and PD-L1 programmed death-ligand 1.

Examination of formalin-fixed, paraffin-embedded (FFPE) tumor tissue from the resection showed an inverse correlation between tumor 2-HG and tumor-infiltrating CD3^+^ and CD8^+^ T cells (Fig. 2g), and again showed an association between 2-HG suppression and upregulation of antigen presentation and the IFN pathways (Extended Data Fig. 6).

**Extended Data Fig. 7:**
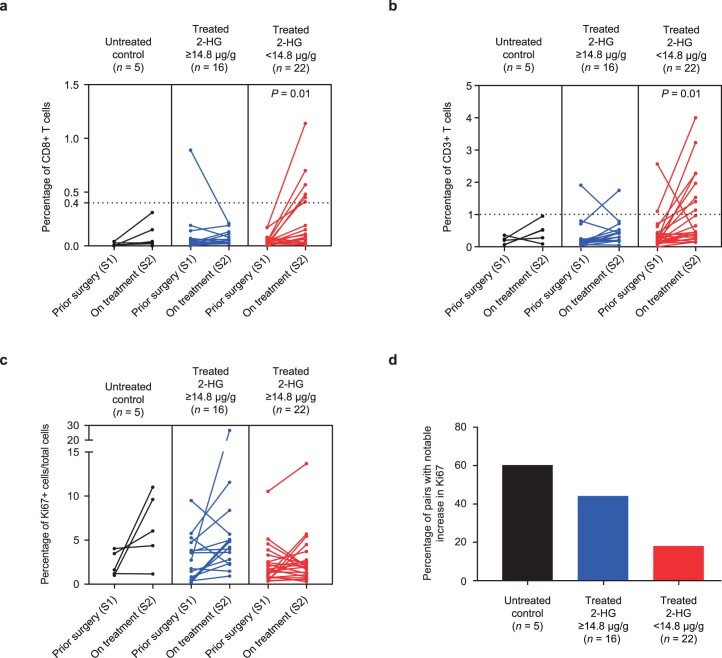
Analysis of CD3 + and CD8 + T Cells, and Ki-67+ cells, in matched surgery 1 (archival)/surgery 2 (on treatment) pairs. **a–c**, Patient-matched tumor-infiltrating CD8 + (panel **a**) and CD3 + (panel **b**) T-cell densities (Student’s two-sided t test), and Ki-67–positive cells (panel **c**) comparing prior surgery and on-treatment tumors. The upper 95% confidence interval of the 2-HG concentrations in wild-type isocitrate dehydrogenase tumors is 14.8 µg/g, as shown in Fig. 2a. On-treatment tumors with 2-HG levels less than 14.8 µg/g are categorized as tumors with more complete 2-HG suppression. **d**, Notable increase refers to an increase of % of Ki67+ cells by more than 2. 2-HG denotes D-2-hydroxyglutarate, S1 matched-pair analysis from surgery 1 (archival tumor tissue from prior surgery), and S2 matched-pair analysis from surgery 2 (on-treatment surgery).

Matched-pair analysis from surgery 1 (archival tumor tissue from previous surgery) and surgery 2 (on-treatment surgery) suggested that more complete tumor 2-HG suppression was required to promote tumor infiltration with CD3^+^/CD8^+^ T cells (Extended Data Fig. 7a,b) and inhibit tumor cell proliferation (Extended Data Fig. 7c,d).

### Preliminary assessment of antitumor activity

**Fig. 3:**
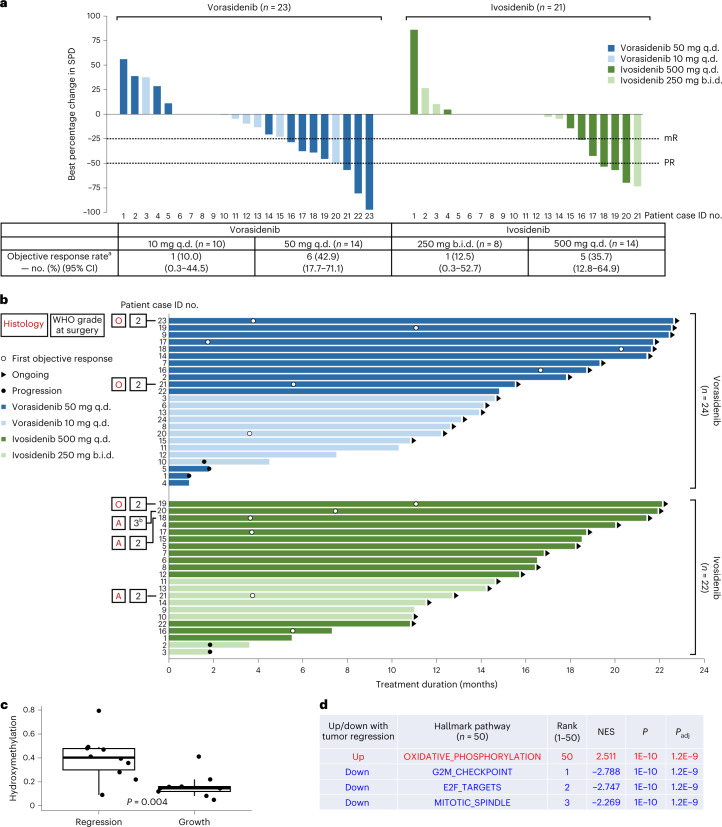
Postoperative tumor response assessment and treatment duration. **a**, The best percentage change in SPD compared with postoperative baseline MRI and the overall ORRs by assigned treatment. Eleven patients had an on-study gross total resection without residual disease and were considered to have best response of stable disease as long as disease progression had not been documented. Patient 22 in the vorasidenib group had a >50% reduction in tumor size compared with baseline that was not confirmed and was therefore categorized as stable disease. Two patients were not included in the plot (patient 24 in the vorasidenib group and patient 22 in the ivosidenib group) owing to changes in selected target lesions measured at one or more postoperative time points that affected best percentage change assessment. ^a^Complete response, PR or mR. **b**, Postoperative treatment duration by treatment group. The histology at the time of initial diagnosis and the WHO grade based on pathology of the on-study resected tumor are provided for six patients who achieved a PR after postoperative treatment with vorasidenib (*n* = 2) or ivosidenib (*n* = 4), according to the investigator’s assessment of response using RANO-LGG. ^b^The tumor grade for this patient changed from grade 2 at screening to grade 3 at surgery. **c**, On-treatment DNA hydroxymethylation levels in the tumor regression (*n* = 12) and tumor growth (*n* = 9) groups. Box plot: the horizontal lines denote the 25th, 50th and 75th percentiles and the upper/lower whiskers extend from the hinge to the largest/smallest value that is within 1.5 × the interquartile range from the hinge; the two-sided *P* value was generated using Student’s *t*-test. **d**, Modulation of selected molecular pathways comparing the tumor regression and tumor growth groups; *P* values adjusted for multiplicity as described in Methods. A, astrocytoma; O, oligodendroglioma; SPD, sum of products of tumor lesion diameters.

Investigator-assessed tumor response showed a decrease in tumor size after postoperative treatment with either vorasidenib or ivosidenib (Fig. 3a). Patients without residual disease (*n* = 11) were considered to have the best response of stable disease if disease progression had not been documented. The objective response rate (ORR) for vorasidenib 50 mg q.d. was 42.9% (95% confidence interval (CI), 17.7−71.1), including two partial responses (PRs) and four minor responses (mRs), and 10.0% (95% CI, 0.3−44.5) for vorasidenib 10 mg q.d. (one mR). The ORR for ivosidenib 500 mg q.d. was 35.7% (95% CI, 12.8−64.9), including three PRs and two mRs, and 12.5% (95% CI, 0.3 − 52.7) for ivosidenib 250 mg b.i.d. (one PR) (Supplementary Table 5). The median postoperative treatment duration was 14.3 months (range 0.9–22.6 months) for vorasidenib and 15.1 months (range 1.8–22.1 months) for ivosidenib (Fig. 3b).

**Extended Data Fig. 8:**
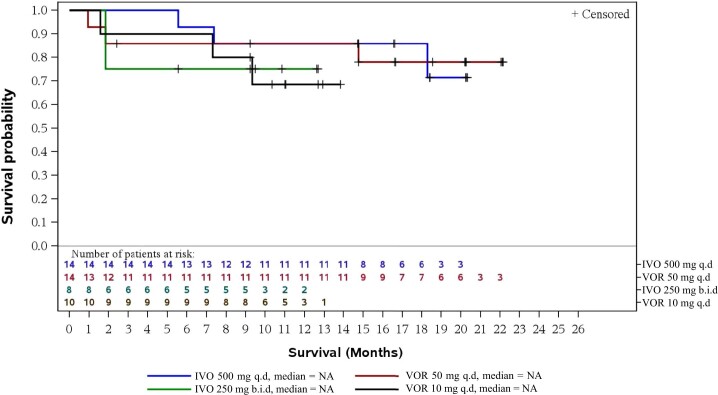
Kaplan-Meier plot of progression-free survival (post-surgery), efficacy analysis set, as of April 29, 2020. B.i.d. denotes twice daily, IVO ivosidenib, q.d. once daily, and VOR vorasidenib.

At the time of manuscript submission, fewer than half the 32 patients who remained on treatment as of the data cutoff date (29 April 2020) have discontinued treatment and the progression-free survival data continue to mature. Preliminary Kaplan–Meier curves of progression-free survival are shown in Extended Data Fig. 8.

**Extended Data Fig. 9:**
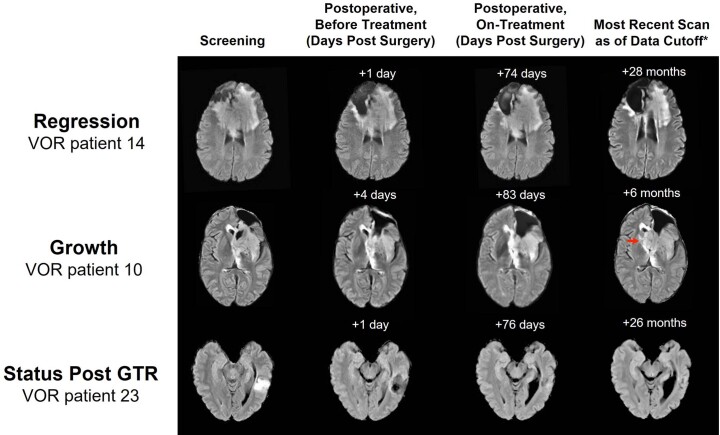
Exploratory analysis of molecular changes in resected tumors and clinical response. *As of April 29, 2020. GTR denotes gross total resection, and VOR vorasidenib.

**Extended Data Fig. 10:**
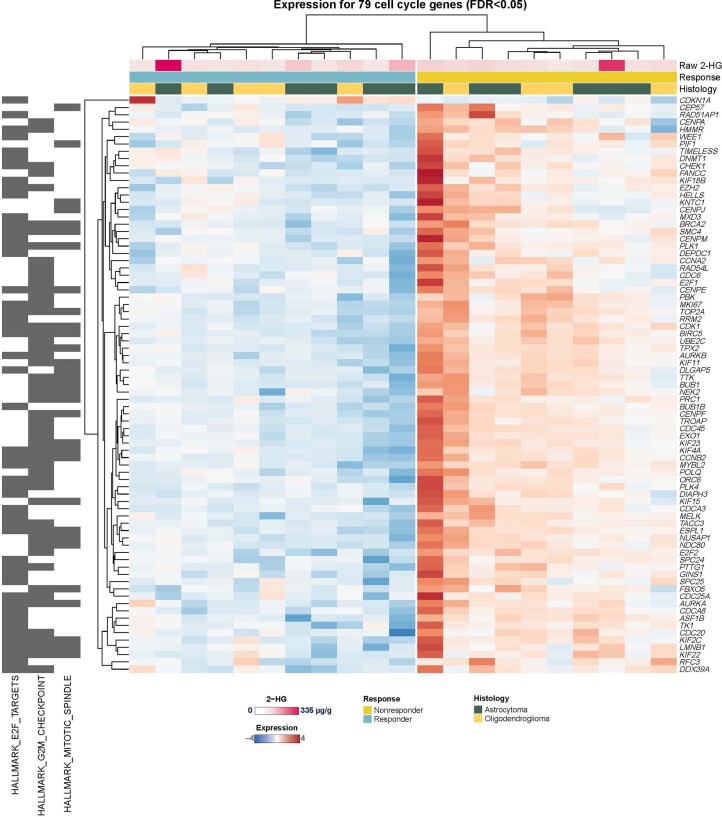
Unsupervised clustering of tumor response–associated genes in hallmark cell cycle–related pathways. 2-HG denotes D-2-hydroxyglutarate, and FDR false discovery rate.

We also examined the relationship between the molecular changes in on-treatment biopsies and subsequent radiographic response. This exploratory post-hoc analysis included a manual consensus review of all MR images by multiple investigators and excluded patients whose tumors neither grew during drug treatment nor could be assessed for tumor regression owing to a previous complete tumor resection (Extended Data Fig. 9). Tumor regression was associated with high tumor DNA 5hmC content (Fig. 3c) and reduced expression of cell-cycle-associated genes (Fig. 3d, Extended Data Fig. 10 and Supplementary Table 6) in the on-treatment biopsies.

## Discussion

*IDH* mutations are believed to play a prominent role in the development of glioma, but the role of the mutant enzyme in recurrent glioma is currently unclear. Our data demonstrate that the m*IDH* enzyme remains active in recurrent disease, because 2-HG reduction was associated with reduced tumor cell proliferation, increased DNA 5hmC content (mediated by TET 5mC hydroxylase activity)^23^ and a reversal of gene expression programs typically associated with *IDH* mutations in LGGs. Although a net loss of malignant glioma cells might explain a loss of proneural transcripts, this would not account for the specific upregulation of mesenchymal transcripts and increase in cellular differentiation markers that we observed.

Our results point toward opportunities for combination therapies with *IDH* inhibitors in glioma. The association between radiographic tumor response and increased DNA 5hmC content, for example, suggests that DNA methylation is an important contributor to tumor maintenance by the m*IDH* enzyme in glioma, reminiscent of the contribution of TET enzymes in m*IDH*-associated tumorigenesis in other cancers^28^. This raises the hypothesis that DNA-hypomethylating agents, such as the DNA methyltransferase inhibitor azacitidine, might augment the antitumor activity of *IDH* inhibitors, as has recently been shown in acute myeloid leukemia^29,30^. Our observation that 2-HG reduction was associated with the induction of genes associated with antitumor immunity and a modest increase in tumor infiltration with CD8^+^ T cells is consistent with an immunosuppressive effect of 2-HG on the glioma microenvironment, as suggested by previous studies^31,32^, and raises the intriguing possibility that inhibition of the mutant enzyme might synergize with other strategies to promote antitumor immunity against m*IDH* gliomas.

Surgical window-of-opportunity trials typically rely on the comparison of matched pre-treatment and on-treatment biopsy pairs from the same patients. It was not feasible for patients enrolled in our current study to undergo a pre-treatment biopsy because we could not ensure that sufficient tumor tissue could be collected through a needle biopsy for 2-HG analysis and key related molecular studies (for example, RNA-sequencing (RNA-seq) or 5hmC analyses) and complications from this procedure could have resulted in a delay of the planned tumor resection. Our comparisons of immunohistochemistry (IHC) results (for example, Ki-67, immune cell infiltration) between on-study tumor tissue and tumor tissue collected during the original diagnostic tumor resection have to be interpreted with caution because many patients received chemotherapy or radiation in the interval between the two surgeries, and these tumor samples were often collected and stored under different conditions. Given these limitations, we included a large number of external untreated control tumors with a borrowing methodology in our primary analysis. The integration of internal and external controls in our clinical trial design may serve as a template for future side-by-side comparisons of investigational agents with similar mechanisms of action for brain tumor patients.

Preliminary indications of clinical activity in our study must be interpreted with caution because the progression-free survival data continue to mature and follow-up time is short for LGGs. A daily dose of vorasidenib 50 mg showed the most consistent inhibition of the mutant enzyme and the greatest preliminary antitumor activity. Based on the current data, vorasidenib (50 mg q.d. of the uncoated tablet formulation used in the present study) was selected for the initiation of the ongoing global phase 3 INDIGO study in grade 2 m*IDH* nonenhancing glioma (ClinicalTrials.gov, NCT04164901; a coated-tablet formulation was later introduced into the INDIGO study at a dose exposure equivalent of 40 mg q.d.). Of note, ivosidenib showed considerably lower CNS penetration than vorasidenib, in line with preclinical studies, but nevertheless reached adequate tumor concentrations to inhibit the m*IDH* enzyme in patients owing to its high plasma exposure. The latter observation highlights that even drugs with low CNS penetrance may warrant a detailed pharmacokinetic/PD evaluation before excluding them from further development for CNS tumors.

In conclusion, our study established the clinical, PD and translational rationale to select a late-stage molecule to target one of the most prevalent mutations in LGGs and provides a rich data resource to advance our understanding of the role of *IDH* mutations and the mechanism of action of *IDH* inhibitors in recurrent gliomas.

## Methods

### Trial design and oversight

This is a randomized, controlled, multicenter, open-label, perioperative study of vorasidenib and ivosidenib in recurrent, nonenhancing m*IDH1* LGGs (ClinicalTrials.gov, NCT03343197). The randomization schedule was generated by an independent statistical group and randomization assignment was implemented by an interactive web response system. Additional details are provided in the study protocol and statistical analysis plan.

The study was conducted according to the International Council on Harmonisation of Good Clinical Practice guidelines and the principles of the Declaration of Helsinki. All patients provided written informed consent before screening and enrollment.

### Patients

Key eligibility criteria included patients with recurrent, CNS WHO 2016 grade 2/3, m*IDH1*-R132H oligodendroglioma or astrocytoma who were surgical candidates. Additional eligibility criteria included age ≥18 years, adequate hepatic and renal function, a Karnofsky performance status score ≥60%, no previous *IDH* inhibitor treatment, ≥6 months since any radiation and measurable nonenhancing lesion by central radiology review. Patients were recruited by the authors at Memorial Sloan Kettering Cancer Center (by I.K.M., *n* = 13), University of California, Los Angeles Medical Center (by T.F.C., *n* = 10), University of Texas Southwestern (by E.A.M., *n* = 8), University of California, San Francisco Division of Neuro-Oncology (by J.W.T., *n* = 7), Dana Farber Cancer Institute (by P.W.Y., *n* = 6), Massachusetts General Hospital (by I.A.-R., *n* = 3) and Duke University Medical Center (by K.B.P., *n* = 2). The study protocol was approved by the institutional review board/independent ethics committee at each of these study locations. Participants’ sex was assigned by the site and no sex- or gender-based analyses were performed because these would have been post hoc and insufficiently powered to enable meaningful conclusions.

### Outcomes

The primary end-point of 2-HG concentration in resected tumors was evaluated by comparing concentrations in patients with m*IDH1* glioma treated with vorasidenib or ivosidenib with concentrations in tumors from untreated on-study patients (internal contemporaneous control) and additional tumors from untreated patients with WT *IDH* (*n* = 15) and m*IDH1* (*n* = 61) glioma (external control using previously banked tumor samples).

Secondary end-points included safety, tumor and plasma pharmacokinetics, and preliminary clinical activity. Exploratory end-points included IDH pathway-related molecular and cellular changes that correlated with 2-HG and radiographic response, where feasible.

Safety was assessed by monitoring all AEs from the time of consent through 28 (+ ≤5) d after the last dose using the National Cancer Institute Common Terminology Criteria for Adverse Events, v.4.03 (ref. ^33^).

Tumor measurements were collected using a standardized MRI protocol at screening and surgery. A brain MR image was collected postoperatively as the new baseline before resuming treatment. Additional brain MR images were collected every 56 (±2) d thereafter (that is, while receiving study treatment) and at the end of treatment. Antitumor activity was assessed by the investigator using the Response Assessment in Neuro-Oncology criteria for low-grade glioma (RANO-LGG)^34^.

### Patient sample collection

Tumor samples were collected and immediately processed for all clinical trial participants. The tissue was immediately split into two parts: the first sample was snap frozen in liquid nitrogen and the second sample was processed into an FFPE block. Pre-treatment, archival FFPE slides were also collected whenever possible and included in selected biomarker analyses (Extended Data Fig. 1). Concentrations of 2-HG and vorasidenib or ivosidenib were measured in tumor and plasma using liquid chromatography with tandem mass spectrometry (LC–MS/MS). Frozen tumor tissue was analyzed for tumor content and cellularity (hematoxylin and eosin (H&E) staining), DNA alterations, genome-wide RNA expression (RNA-seq) and DNA methylation. FFPE tumor tissue was analyzed for tumor content (H&E staining), IHC and expression profiling of selected genes (Extended Data Fig. 1). Patients who missed two or more doses in two weeks before surgery, or for whom no or inadequate m*IDH*-containing tumor tissue was received, were replaced in the 2-HG analysis.

### DNA-seq

DNA-seq of archival FFPE slides and frozen surgical samples was performed by next-generation sequencing using the ACE Extended Cancer Panel (Personalis).

### DNA methylation profiling

To determine the levels of 5hmC, DNA extracted from frozen surgical samples were digested with DNA degradase (Zymo Research) to generate single nucleosides. LC–MS/MS was used to quantify 5hmC, 5mC and cytosine (C). The percentage of 5hmC was calculated as the ratio 5hmC:C.

### RNA expression profiling

Transcriptional profiling (RNA-seq) of frozen surgical samples was conducted using the ACE Research Transcriptome assay (Personalis). Paired-end reads in FASTQ format were aligned to the human genome (GRCh38, release 85 (ref. ^35^)) with HISAT, v.2.0.5 (ref. ^36^). SAM-to-BAM conversion and sorting were performed using Samtools v.1.4 (ref. ^37^). Transcript assembly with RefSeq annotation in GTF format and gene abundance estimation were carried out using StringTie v.1.3.3b and the built-in prep_DE.py Python script^38^, producing gene-level raw count expression values as well as transcripts per million (that is, counts corrected for gene length and sequencing depth). All subsequent analyses were conducted in the R environment v.4.1 (ref. ^39^).

To evaluate the association between gene expression and 2-HG concentrations, differential expression analysis was conducted on raw count expression values for a complete set of 33,121 genes. A negative binomial generalized linear model, as implemented in the DESeq2 v.1.24.0R package^40^, was fit to identify genes with an expression associated with *z*-scored log_10_(transformed 2-HG levels), after correcting for histology and treatment status. *P* values were adjusted for multiple testing using the false discovery rate (FDR)/Benjamini–Hochberg method^41^. For pathway enrichment analysis, genes were first sorted by the significance and the direction of their association with 2-HG, according to the formula:

$${\mathrm{rank}} = -{\mathrm{log}}_{10}\left( {{\mathrm{unadjusted}}\,P\,{\mathrm{value}}} \right) \times {\mathrm{sign}}\left( {{\mathrm{log}}_2({\mathrm{FC}})} \right)$$

where log_2_(FC) represents log_2_(transformed moderated fold-changes)^42^. The ranking was then used as input in gene set enrichment analysis (GSEA)^43,44^. Enrichment scores were calculated using the fast GSEA (FGSEA) v.1.12.0R package^45^ against MSigDB curated gene set (C2) and Hallmark pathways (H), with gene-set sizes ranging from 15 to 500, using 1,000 permutations. Pathways with a positive normalized enrichment score (NES) contained genes downregulated with the suppression of 2-HG. Pathways with a negative NES contained genes upregulated with the suppression of 2-HG.

To visually demonstrate the relationship between gene expression and 2-HG concentration, a heatmap of *z*-scored variance stabilization-transformed (VST) values was generated for the differentially expressed genes belonging to select top-scoring, significantly enriched pathways using pheatmap v.1.0.12 (ref. ^46^). Complete linkage hierarchical clustering method with Euclidean distance was used to cluster the genes in rows and cluster the samples in columns; the two topmost column clusters were considered to represent patients with high 2-HG and decreased gene expression and patients with low 2-HG and increased gene expression.

To identify genes that are associated with *IDH* mutations in LGGs, we used HTSeq count data from 413 WT and 94 m*IDH* LGG primary tumor samples from the Cancer Genome Atlas-LGG transcriptome profiling dataset^47,48^. To remove genes expressed at low levels, we converted raw count expression values to counts per million (c.p.m.), using the edgeR v.26.4 (ref. ^49^) R package to account for sequencing depth. We retained genes with c.p.m. ≥ 0.76 in ≥94 samples. Of 56,404 genes, 18,416 passed the filter and were used in subsequent analyses. Gene expression analysis was conducted on raw count expression values. We used the DESeq2 package to fit a negative binomial generalized linear model to identify genes expressed differentially in m*IDH* and WT samples, after correcting for 1p19q codeletion status. *P* values were adjusted for multiple comparisons using FDR.

To identify genes differentially expressed between samples from patients whose tumors responded to treatment (tumor regression) and those whose tumors did not (continued tumor growth), gene expression analysis was conducted on raw count expression values for a complete set of 33,121 genes. We used DESeq2 v.1.24.0 to fit a negative binomial generalized linear model, correcting for histology. *P* values were FDR corrected. For pathway enrichment analysis, genes were first sorted by the significance and direction of their association with tumor response. The ranking was then used to calculate enrichment scores in the FGSEA package^45^ against MSigDB Hallmark pathways (H) using the same parameters as detailed above. Pathways with a positive NES contained genes upregulated with tumor regression. Pathways with a negative NES contained genes downregulated with tumor regression.

To visually demonstrate the relationship between gene expression and tumor response, a heatmap of *z*-scored VST values was generated for the differentially expressed genes belonging to select significantly enriched pathways using the pheatmap v.1.0.12 (ref. ^46^) R package. Complete linkage hierarchical (semisupervised) clustering method with Euclidean distance was used to cluster samples in columns (in the two tumor response groups separately) and cluster genes in rows.

NanoString gene expression assay was performed on RNA extracted from FFPE tumor biopsies after macrodissection to enrich tumor content and then scanned using the nCounter Digital Analyzer as per the manufacturer’s instructions (NanoString Technologies). Gene expression was analyzed using nSolver software 4.0 (NanoString Technologies) and the expression levels of each gene were normalized to those of control genes.

### Immunohistochemistry

IHC for Ki-67, CD3 and CD8 was performed by Mosaic Laboratories and quantification derived from an annotation, including all tumor and intervening stroma in the tumor nest. CD3 (mouse clone LN10) immunoglobulin (Ig)G1 antibody (catalog no. NCL-L-CD3-565) was purchased from Leica Biosystems. The CD8 (mouse clone C8/144B) IgG1κ antibody (catalog no. M7103) and the Ki-67 (mouse clone MIB-1) IgG1κ antibody (catalog no. M7240) were purchased from Dako. Antibodies were diluted per the manufacturer’s instructions. All antibodies were stored at 2–8 °C.

### Statistical analysis

Unless otherwise specified, graphs and statistical analyses were performed using GraphPad Prism.

All randomized patients with m*IDH1*-R132H glioma, as confirmed by IHC with an antibody specific to m*IDH1*-R132H or DNA-seq, were included in the primary end-point analysis. A Bayesian hierarchical normal model^50^ was used to compare 2-HG concentrations, on a log_10_ scale, in evaluable treated and untreated control tumors. The model was used to dynamically borrow 2-HG concentration from externally banked, untreated, frozen control samples and the enrolled, untreated control patients. Independent, noninformative, normal distributions and inverse-gamma distributions were used for scale and variance parameters, respectively. The Markov Chain Monte Carlo Gibbs sampling^51^ was used to estimate the posterior distributions of unknown parameters. The posterior mean and 95% CrI of the treatment effect on the 2-HG percentage reduction relative to untreated control groups were provided for each treatment arm. The sample size was determined using extensive simulations on Bayesian analysis for the primary end-point; the sample size achieves approximately 94% probability of detecting that the 2-HG concentrations of the treated group are less than those of the untreated group.

The safety analysis set comprised randomized patients who received at least one dose of vorasidenib or ivosidenib either pre- or postoperatively. Baseline disease characteristics and safety data were summarized by treatment and dose.

Concentrations of vorasidenib and ivosidenib in tumor and plasma were reported as geometric mean and tumor:plasma ratios summarized by treatment and dose. Association of 2-HG with 5hmC, Ki-67 and CD3^+^/CD8^+^ T cells was assessed using simple linear regression. Student’s *t*-test was used to compare two groups. All reported *P* values are two sided.

### Reporting summary

Further information on research design is available in the Nature Portfolio Reporting Summary linked to this article.

## Online content

Any methods, additional references, Nature Portfolio reporting summaries, source data, extended data, supplementary information, acknowledgements, peer review information; details of author contributions and competing interests; and statements of data and code availability are available at 10.1038/s41591-022-02141-2.

## Supplementary information

Supplementary Information Supplementary Tables 1, 2 and 5, and captions for Supplementary Tables 3, 4 and 6.

Reporting Summary

Supplementary Tables 3, 4 and 6 Supplementary Table 3. 2-HG suppression pathways (C2). See tab 1 ‘Suppl T3 2-HG supp pathways C2’. Supplementary Table 4. 2-HG suppression pathways (Hallmark). See tab 2 ‘Suppl T4 2-HG suppr paths Hallm’. Supplementary Table 6. Response analysis (Hallmark). See tab 3 ‘Suppl T6 Response analysis Hall’. For each: *P* values adjusted for multiplicity as described in the Methods.

## Data Availability

We used the publicly available GRCh38, release 85 human genome (https://www.ncbi.nlm.nih.gov/assembly/GCF_000001405.26/) in our analyses. The RNA-seq data generated in the present study are available with the dbGAP accession no. phs003148.v1.p1. Study-level clinical data from this study (including the protocol) will be made available upon reasonable request from a qualified medical or scientific professional for the specific purpose laid out in that request and may include deidentified individual participant data. The data for this request will be available after a data access agreement has been signed. Please send your data-sharing request to https://clinicaltrials.servier.com/data-request-portal. Access to patient-level data depends on a number of constraints, such as the year the study was performed and an anonymization procedure. Requests are reviewed by a qualified panel of Servier experts and, if necessary, by an independent review board and decisions will be communicated within three months, as detailed on the website.

## References

[1] van den Bent MJ, Smits M, Kros JM, Chang SM (2017). Diffuse infiltrating oligodendroglioma and astrocytoma. J. Clin. Oncol..

[2] Wen PY (2020). Glioblastoma in adults: a Society for Neuro-Oncology (SNO) and European Society of Neuro-Oncology (EANO) consensus review on current management and future directions. Neuro-Oncology.

[3] Louis DN (2016). The 2016 World Health Organization classification of tumors of the central nervous system: a summary. Acta Neuropathol..

[4] Hardee ME, Zagzag D (2012). Mechanisms of glioma-associated neovascularization. Am. J. Pathol..

[5] Claus EB (2015). Survival and low-grade glioma: the emergence of genetic information. Neurosurg. Focus.

[6] Klein M (2002). Effect of radiotherapy and other treatment-related factors on mid-term to long-term cognitive sequelae in low-grade gliomas: a comparative study. Lancet.

[7] Yan H (2009). IDH1 and IDH2 mutations in gliomas. N. Engl. J. Med..

[8] Dang L (2009). Cancer-associated IDH1 mutations produce 2-hydroxyglutarate. Nature.

[9] Ward PS (2010). The common feature of leukemia-associated IDH1 and IDH2 mutations is a neomorphic enzyme activity converting alpha-ketoglutarate to 2-hydroxyglutarate. Cancer Cell.

[10] Xu W (2011). Oncometabolite 2-hydroxyglutarate is a competitive inhibitor of α-ketoglutarate-dependent dioxygenases. Cancer Cell.

[11] Brat DJ, Cancer Genome Atlas Research Network (2015). Comprehensive, integrative genomic analysis of diffuse lower-grade gliomas. N. Engl. J. Med..

[12] Losman JA, Kaelin WG (2013). What a difference a hydroxyl makes: mutant IDH, (*R*)-2-hydroxyglutarate, and cancer. Genes Dev..

[13] Noushmehr H (2010). Identification of a CpG island methylator phenotype that defines a distinct subgroup of glioma. Cancer Cell.

[14] Turcan S (2012). IDH1 mutation is sufficient to establish the glioma hypermethylator phenotype. Nature.

[15] TIBSOVO (ivosidenib). *Highlights of Prescribing Information* (Servier Pharmaceuticals LLC, 2022).

[16] Mellinghoff IK (2020). Ivosidenib in isocitrate dehydrogenase 1-mutated advanced glioma. J. Clin. Oncol..

[17] Tap WD (2020). Phase I study of the mutant IDH1 inhibitor ivosidenib: safety and clinical activity in patients with advanced chondrosarcoma. J. Clin. Oncol..

[18] Konteatis Z (2020). Vorasidenib (AG-881): a first-in-class, brain-penetrant dual inhibitor of mutant IDH1 and 2 for treatment of glioma. ACS Med. Chem. Lett..

[19] Mellinghoff IK (2021). Vorasidenib, a dual inhibitor of mutant IDH1/2, in recurrent or progressive glioma; results of a first-in-human phase I trial. Clin. Cancer Res..

[20] Popovici-Muller J (2018). Discovery of AG-120 (ivosidenib): a first-in-class mutant IDH1 inhibitor for the treatment of IDH1 mutant cancers. ACS Med. Chem. Lett..

[21] Liberzon A (2011). Molecular signatures database (MSigDB) 3.0. Bioinformatics.

[22] Verhaak RG (2010). Integrated genomic analysis identifies clinically relevant subtypes of glioblastoma characterized by abnormalities in PDGFRA, IDH1, EGFR, and NF1. Cancer Cell.

[23] Figueroa ME (2010). Leukemic IDH1 and IDH2 mutations result in a hypermethylation phenotype, disrupt TET2 function, and impair hematopoietic differentiation. Cancer Cell.

[24] Lu C (2012). IDH mutation impairs histone demethylation and results in a block to cell differentiation. Nature.

[25] Saha SK (2014). Mutant IDH inhibits HNF-4α to block hepatocyte differentiation and promote biliary cancer. Nature.

[26] Rohle D (2013). An inhibitor of mutant IDH1 delays growth and promotes differentiation of glioma cells. Science.

[27] Wang F (2013). Targeted inhibition of mutant IDH2 in leukemia cells induces cellular differentiation. Science.

[28] Losman JA (2013). (*R*)-2-Hydroxyglutarate is sufficient to promote leukemogenesis and its effects are reversible. Science.

[29] DiNardo CD (2021). Enasidenib plus azacitidine versus azacitidine alone in patients with newly diagnosed, mutant-IDH2 acute myeloid leukaemia (AG221-AML-005): a single-arm, phase 1b and randomised, phase 2 trial. Lancet Oncol..

[30] Montesinos P (2022). Ivosidenib and azacitidine in IDH1-mutated acute myeloid leukemia. N. Engl. J. Med..

[31] Kohanbash G (2017). Isocitrate dehydrogenase mutations suppress STAT1 and CD8+ T cell accumulation in gliomas. J. Clin. Invest..

[32] Bunse L (2018). Suppression of antitumor T cell immunity by the oncometabolite (*R*)-2-hydroxyglutarate. Nat. Med..

[33] Common Terminology Criteria for Adverse Events (CTCAE), v.4.03. *National Cancer Institute* https://ctep.cancer.gov/protocolDevelopment/electronic_applications/ctc.htm (2006).

[34] van den Bent MJ (2011). Response assessment in neuro-oncology (a report of the RANO group): assessment of outcome in trials of diffuse low-grade gliomas. Lancet Oncol..

[35] Zerbino DR (2018). Ensembl 2018. Nucleic Acids Res..

[36] Kim D, Langmead B, Salzberg SL (2015). HISAT: a fast spliced aligner with low memory requirements. Nat. Methods.

[37] Li H (2009). The Sequence Alignment/Map format and SAMtools. Bioinformatics.

[38] Pertea M (2015). StringTie enables improved reconstruction of a transcriptome from RNA-seq reads. Nat. Biotechnol..

[39] Gentleman, R. et al. *R: A Language and Environment for Statistical Computing* (R Core Team, 2021); https://www.r-project.org

[40] Love MI, Huber W, Anders S (2014). Moderated estimation of fold change and dispersion for RNA-seq data with DESeq2. Genome Biol..

[41] Benjamini Y, Hochberg Y (1995). Controlling the false discovery rate: a practical and powerful approach to multiple testing. J. R. Stat. Soc. Ser. B Stat. Methodol..

[42] Zhu A, Ibrahim JG, Love MI (2019). Heavy-tailed prior distributions for sequence count data: removing the noise and preserving large differences. Bioinformatics.

[43] Subramanian A (2005). Gene set enrichment analysis: a knowledge-based approach for interpreting genome-wide expression profiles. Proc. Natl Acad. Sci. USA.

[44] Mootha VK (2003). PGC-1alpha-responsive genes involved in oxidative phosphorylation are coordinately downregulated in human diabetes. Nat. Genet..

[45] Korotkevich, G. et al. Fast gene set enrichment analysis. Preprint at *bioRxiv*10.1101/060012 (2021).

[46] Kolde, R. pheatmap: pretty heatmaps. R version 1.0.12 https://CRAN.R-project.org/package=pheatmap (2019).

[47] Pedano, N. et al. Radiology data from The Cancer Genome Atlas Low Grade Glioma [TCGA-LGG] collection. *The Cancer Imaging Archive* (2015); https://www.cancerimagingarchive.net/

[48] *The Cancer Genome Atlas Program* (National Cancer Institute at the National Institutes of Health, accessed 11 February 2022); http://cancergenome.nih.gov

[49] Robinson MD, McCarthy DJ, Smyth G (2010). K. edgeR: a Bioconductor package for differential expression analysis of digital gene expression data. Bioinformatics.

[50] Viele K (2014). Use of historical control data for assessing treatment effects in clinical trials. Pharm. Stat..

[51] Plummer, M. JAGS: a program for analysis of Bayesian graphical models using Gibbs sampling. In *Proc. of the 3rd International Workshop on Distributed Statistical Computing* (Hornik, K. et al eds) March 20-22, 2003, Vienna, Austria; https://www.r-project.org/conferences/DSC-2003/Proceedings/Plummer.pdf

