# Author Correction: Vorasidenib and ivosidenib in IDH1-mutant low-grade glioma: a randomized, perioperative phase 1 trial

**DOI:** 10.1038/s41591-023-02473-7

**Published:** 2023-07-03

**Authors:** Ingo K. Mellinghoff, Min Lu, Patrick Y. Wen, Jennie W. Taylor, Elizabeth A. Maher, Isabel Arrillaga-Romany, Katherine B. Peters, Benjamin M. Ellingson, Marc K. Rosenblum, Saewon Chun, Kha Le, Ania Tassinari, Sung Choe, Youssef Toubouti, Steven Schoenfeld, Shuchi S. Pandya, Islam Hassan, Lori Steelman, Jennifer L. Clarke, Timothy F. Cloughesy

**Affiliations:** 1 Memorial Sloan Kettering Cancer Center, New York, NY USA; 2 Agios Pharmaceuticals, Cambridge, MA USA; 3 Dana-Farber Cancer Institute, Boston, MA USA; 4 University of California San Francisco, San Francisco, CA USA; 5 University of Texas Southwestern Medical Center, Dallas, TX USA; 6 Massachusetts General Hospital, Harvard Medical School, Boston, MA USA; 7 Duke University Medical Center, Durham, NC USA; 8 University of California, Los Angeles, Los Angeles, CA USA; 9 California University of Science and Medicine, Colton, CA USA; 10 Aligos Therapeutics, South San Francisco, CA USA; 11 Servier Pharmaceuticals LLC, Boston, MA USA; 12 Present Address: Mersana Therapeutics, Cambridge, MA USA; 13 Present Address: Sage Therapeutics, Cambridge, MA USA

**Keywords:** CNS cancer, Drug development

Correction to: *Nature Medicine* 10.1038/s41591-022-02141-2. Published online 23 February 2023.

This paper was originally published under a standard Springer Nature license (© The Author(s), under exclusive licence to Springer Nature America, Inc.). It is now available as an open-access paper under a Creative Commons Attribution 4.0 International license, © The Author(s). The error has been corrected in the HTML and PDF versions of the article.

