# Supplementary material for: Vorasidenib and ivosidenib in IDH1-mutant low-grade glioma: a randomized, perioperative phase 1 trial
Source: Nat Med. 2023 Feb 23;29(3):615–22. doi: 10.1038/s41591-022-02141-2 (PMC10313524; doi:10.1038/s41591-022-02141-2)
Supplement: Supplementary file 1 — Supplementary Tables 1, 2 and 5, and captions for Supplementary Tables 3, 4 and 6. [file 41591_2022_2141_MOESM1_ESM.pdf]

nature medicine

Article https://doi.org/10.1038/s41591-022-02141-2

Vorasidenib and ivosidenib in IDH1-mutant low-grade glioma: a randomized, perioperative phase 1 trial

In the format provided by the authors and unedited

Supplementary tables

Supplementary Table 1. Summary of pharmacokinetic parameters of vorasidenib and ivosidenib in tumor and plasma.*

**Time of Surgery Sample**	**Tumor — ng/g**				**Plasma C_{avg} — ng/ml**			
	**Vorasidenib**		**Ivosidenib**		**Vorasidenib**		**Ivosidenib**	
	**50 mg QD (N=9)**	**10 mg QD (N=9)**	**500 mg QD (N=11)**	**250 mg BID (N=9)**	**50 mg QD (N=10)**	**10 mg QD (N=9)**	**500 mg QD (N=11)**	**250 mg BID (N=9)**
Geometric mean (range) drug concentration	110 (59.8–190.0)	66.7 (33.2–113.0)	233 (105.0–604.0)	278 (109.0–594.0)	70 (31.6–144.0)	22.4 (11.2–34.9)	2618 (1762.0–3369.0)	2435 (1597.0–3829.0)
Mean±SD brain-to-plasma ratio	1.69±0.55	2.92±1.13†	0.10±0.05	0.13±0.08†				

*BID denotes twice daily, C_{avg} average concentration over the dosing interval, and QD once daily.
†N=8.

Supplementary Table 2. Summary of TEAEs.*

	Vorasidenib			Ivosidenib		
	**50 mg QD (N=14)**	**10 mg QD (N=10)**	**Total (N=24)**	**500 mg QD (N=15)**	**250 mg BID (N=10)**	**Total (N=25)**
All patients — no. (%)†						
Patients with ≥1 TEAE	14 (100)	10 (100)	24 (100)	15 (100)	10 (100)	25 (100)
Patients with treatment-related TEAE	10 (71.4)	4 (40.0)	14 (58.3)	12 (80.0)	5 (50.0)	17 (68.0)
Patients with serious TEAEs	3 (21.4)	1 (10.0)	4 (16.7)	4 (26.7)	1 (10.0)	5 (20.0)
Patients with treatment-related serious TEAE	1 (7.1)	0	1 (4.2)	0	0	0
Patients with ≥1 grade ≥3 TEAE	5 (35.7)	2 (20.0)	7 (29.2)	5 (33.3)	1 (10.0)	6 (24.0)
Patients with ≥1 treatment-related grade ≥3 TEAE	1 (7.1)	1 (10.0)	2 (8.3)	1 (6.7)	0	1 (4.0)
Patients with ≥1 TEAE of special interest‡	2 (14.3)	0	2 (8.3)	0	0	0
Patients with TEAE leading to drug discontinuation	0	0	0	1 (6.7)	0	1 (4.0)
Patients with TEAE leading to drug reduction	1 (7.1)	0	1 (4.2)	0	0	0
Patients with TEAE leading to drug interruption	3 (21.4)	2 (20.0)	5 (20.8)	3 (20.0)	0	3 (12.0)

*BID denotes twice daily, QD once daily, and TEAE treatment-emergent adverse event.
†Includes all patients who received at least one dose of ivosidenib or vorasidenib in the pre- or postoperative treatment period; untreated control patients are categorized according to postoperative treatment. Only TEAEs occurring on or after the first dose of study drug are included.
Any grade 3 or higher QT prolongation event in patients receiving ivosidenib, regardless of seriousness, were reported as a TEAE of special interest. Any grade 2 or worse alanine aminotransferase or aspartate aminotransferase elevation in patients receiving vorasidenib, regardless of seriousness, were reported as a TEAE of special interest.

Supplementary Table 3. 2-HG suppression pathways (C2).

See separate spreadsheet file (Mellinghoff et al Supplemental Tables 3, 4 and 6.xlsx), tab 1

‘Suppl T3 2-HG supp pathways C2’. *P* values adjusted for multiplicity as described in Methods.

NES denotes normalized enrichment score, padj adjusted *P* value, pval *P* value.

Supplementary Table 4. 2-HG suppression pathways (Hallmark).

See separate spreadsheet file (Mellinghoff et al Supplemental Tables 3, 4 and 6.xlsx), tab 2

‘Suppl T4 2-HG suppr paths Hallm’. *P* values adjusted for multiplicity as described in Methods.

NES denotes normalized enrichment score, padj adjusted *P* value, pval *P* value.

Supplementary Table 5. Tumor response by response assessment in neuro-oncology criteria for low-grade gliomas according to the investigator.*

	**Vorasidenib**			**Ivosidenib**		
	**50 mg QD (N=14)**	**10 mg QD (N=10)**	**Total (N=24)**	**500 mg QD (N=14)**	**250 mg BID (N=8)**	**Total (N=22)**
Best overall response — no. (%)						
Complete response	0	0	0	0	0	0
Partial response	2 (14.3)	0	2 (8.3)	3 (21.4)	1 (12.5)	4 (18.2)
Minor response	4 (28.6)	1 (10.0)	5 (20.8)	2 (14.3)	0	2 (9.1)
Stable disease	6 (42.9)	8 (80.0)	14 (58.3)	9 (64.3)	5 (62.5)	14 (63.6)
Progressive disease	2 (14.3)	1 (10.0)	3 (12.5)	0	2 (25.0)	2 (9.1)
Objective response rate† — no. (%) [95% CI]	6 (42.9) [17.7−71.1]	1 (10.0) [0.3−44.5]	7 (29.2) [12.6−51.1]	5 (35.7) [12.8−64.9]	1 (12.5) [0.3−52.7]	6 (27.3) [10.7−50.2]

*BID denotes twice daily, and QD once daily.
†Complete response, partial response, or minor response.

Supplementary Table 6. Response analysis (Hallmark).

See separate spreadsheet file (Mellinghoff et al Supplemental Tables 3, 4 and 6.xlsx), tab 3

‘Suppl T6 Response analysis Hall’. *P* values adjusted for multiplicity as described in Methods.

NES denotes normalized enrichment score, padj adjusted *P* value, pval *P* value.
