# Supplementary material for: Vorasidenib and ivosidenib in IDH1-mutant low-grade glioma: a randomized, perioperative phase 1 trial
Source: Nat Med. 2023 Feb 23;29(3):615–22. doi: 10.1038/s41591-022-02141-2 (PMC10313524; doi:10.1038/s41591-022-02141-2)
Supplement: Supplementary file 2 — Reporting Summary [file 41591_2022_2141_MOESM2_ESM.pdf]

nature portfolio

Corresponding author(s): Ingo K. Mellinghoff

Last updated by author(s): Nov 1, 2022

Reporting Summary

Nature Portfolio wishes to improve the reproducibility of the work that we publish. This form provides structure for consistency and transparency in reporting. For further information on Nature Portfolio policies, see our Editorial Policies and the Editorial Policy Checklist.

Statistics

For all statistical analyses, confirm that the following items are present in the figure legend, table legend, main text, or Methods section.

n/a	Confirmed	
☐	☒	The exact sample size (*n*) for each experimental group/condition, given as a discrete number and unit of measurement
☐	☒	A statement on whether measurements were taken from distinct samples or whether the same sample was measured repeatedly
☐	☒	The statistical test(s) used AND whether they are one- or two-sided<br>*Only common tests should be described solely by name; describe more complex techniques in the Methods section.*
☒	☐	A description of all covariates tested
☐	☒	A description of any assumptions or corrections, such as tests of normality and adjustment for multiple comparisons
☐	☒	A full description of the statistical parameters including central tendency (e.g. means) or other basic estimates (e.g. regression coefficient) AND variation (e.g. standard deviation) or associated estimates of uncertainty (e.g. confidence intervals)
☐	☒	For null hypothesis testing, the test statistic (e.g. *F*, *t*, *r*) with confidence intervals, effect sizes, degrees of freedom and *P* value noted<br>*Give P values as exact values whenever suitable.*
☐	☒	For Bayesian analysis, information on the choice of priors and Markov chain Monte Carlo settings
☒	☐	For hierarchical and complex designs, identification of the appropriate level for tests and full reporting of outcomes
☒	☐	Estimates of effect sizes (e.g. Cohen's *d*, Pearson's *r*), indicating how they were calculated

Our web collection on statistics for biologists contains articles on many of the points above.

Software and code

Policy information about availability of computer code

Data collection	Nanostring gene expression assay was performed on RNA extracted from FFPE tumor biopsies and then scanned using the nCounter® Digital Analyzer
Data analysis	RNA sequencing: Paired-end reads in FASTQ format were aligned to the human genome (GRCh38, release 8535) with HISAT, version 2.0.5. SAM to BAM conversion and sorting was performed using Samtools, version 1.4. Transcript assembly with RefSeq annotation in GTF format and gene abundance estimation were carried out using StringTie, version 1.3.3b, and the built-in prep_DE.py Python script. All subsequent analyses were conducted in the R environment, version 4.1 (R Core Team [2021]).<br>P values were adjusted for multiple testing using the false discovery rate (FDR)/Benjamini-Hochberg method. Enrichment scores were calculated using the FGSEA v1.12.0 R package against MSigDB curated gene set and hallmark pathways. Heatmaps were generated using the pheatmap, version 1.0.12, R package. We used HTSeq count data from The Cancer Genome Atlas-LGG transcriptome profiling dataset. We converted raw count expression values to counts per million using edgeR, version 3.26.4 R package. We used DESeq2 v1.24.0 R package to fit negative binomial generalized linear models. Gene expression was analyzed using nSolver software 4.0.

For manuscripts utilizing custom algorithms or software that are central to the research but not yet described in published literature, software must be made available to editors and reviewers. We strongly encourage code deposition in a community repository (e.g. GitHub). See the Nature Portfolio guidelines for submitting code & software for further information.

Data

Policy information about availability of data

All manuscripts must include a data availability statement. This statement should provide the following information, where applicable:

- Accession codes, unique identifiers, or web links for publicly available datasets
- A description of any restrictions on data availability
- For clinical datasets or third party data, please ensure that the statement adheres to our policy

We used the publicly available GRCh38, release 85 human genome (https://www.ncbi.nlm.nih.gov/assembly/GCF_000001405.26/) in our analyses. The RNA sequencing data generated in this study is available at https://www.ncbi.nlm.nih.gov/bioproject/898324 with the accession number PRJNA898324. Study-level clinical data from this study (including the protocol) will be made available upon reasonable request from a qualified medical or scientific professional for the specific purpose laid out in that request and may include deidentified individual participant data. The data for this request will be available after a data access agreement has been signed. Please send your data sharing request to https://clinicaltrials.servier.com/data-request-portal/. Access to patient-level data depends on a number of constraints, such as the year the study was performed and an anonymization procedure. Requests are reviewed by a qualified panel of Servier experts and if necessary, by an Independent Review Board, and decisions will be communicated within 3 months, as detailed on the website.

Human research participants

Policy information about studies involving human research participants and Sex and Gender in Research.

Reporting on sex and gender	Participants' sex was assigned by the site. Disaggregated individual level data is not reported, and no sex- or gender-based analyses were performed since these would have been post hoc and insufficiently powered to enable meaningful conclusions.
Population characteristics	49 participants were randomized, aged between 19 and 75 years; 33 were men and 16 were women. All had recurrent, nonenhancing mutant IDH1 low-grade glioma. Most patients had WHO grade 2 tumors (43 of 49; 87.8%) based on the most recent pathology before screening. All patients had at least one prior surgery; 24 (49.0%) received prior systemic therapy and 14 (28.6%) received prior radiation therapy.
Recruitment	Participants were recruited by their physicians (the authors). At each of the participating study sites, all glioma patients who met eligibility criteria were offered enrollment into the study
Ethics oversight	Patients were recruited by the authors at Memorial Sloan Kettering Cancer Center (I.K.M., n=13), University of California, Los Angeles Medical Center (T.F.C., n=10), University of Texas Southwestern (E.A.M., n=8), University of California, San Francisco Division of Neuro-Oncology (J.W.T., n=7), Dana Farber Cancer Institute (P.W.Y., n=6), Massachusetts General Hospital (I.A.-R., n=3), and Duke University Medical Center (K.B.P., n=2). The study protocol was approved by the Institutional Review Board/ Independent Ethics Committee at each of these study locations.

Note that full information on the approval of the study protocol must also be provided in the manuscript.

Field-specific reporting

Please select the one below that is the best fit for your research. If you are not sure, read the appropriate sections before making your selection.

☒ Life sciences ☐ Behavioural & social sciences ☐ Ecological, evolutionary & environmental sciences

For a reference copy of the document with all sections, see nature.com/documents/nr-reporting-summary-flat.pdf

Life sciences study design

All studies must disclose on these points even when the disclosure is negative.

Sample size	Extensive statistical simulations were conducted to evaluate the operating characteristics of the design for this study (Appendix 15.9 of the clinical protocol). Based on the results of simulations, assuming 2-HG concentrations between the untreated group and the banked frozen reference samples were similar to allow appropriate borrowing between the two data sources with a standard deviation of 0.28, and the true difference between the first tested dose group and the untreated control group was -1 on log10 scale (90% reduction on original 2-HG concentration scale), 10 patients in the first tested dose group, 5 patients in the untreated control group, and dynamic borrowing from about 25 banked frozen reference samples, the probability that at least a 97.5% probability that 2-HG concentrations of the treated group were less than the untreated group and at least a 70% probability that 2-HG concentrations of the treated group were lower than untreated subjects by at least -0.7 units (which represents 80% reduction on original 2-HG concentration scale) would be 94%. The comparison of the relevant treated group with untreated control group were conducted separately for vorasidenib and ivosidenib. Details about the statistical simulation assumptions are in Appendix 15.9 of the clinical protocol.
Data exclusions	Detailed in manuscript: Nine patients were excluded from the tissue analysis because they did not have enough remaining tissue (N=2), because mIDH1 was not confirmed in resected tissue (N=3), or because they received incorrect drug doses before surgery (N=4).

Replication	Due to limited amount of patient samples, we did not replicate the experiments.
Randomization	Eligible patients were randomized in a 2:2:1 ratio to receive ivosidenib 500 mg QD, vorasidenib 50 mg QD, or to receive no study drug prior to surgery. When both the ivosidenib 250 mg BID and vorasidenib 10 mg QD doses were opened, patients were randomized in a 1:1 ratio to receive either ivosidenib 250 mg BID or vorsidenib 10 mg QD. The randomization scheme was generated by an independent third party.
Blinding	This study was open-label and no placebo was used therefore blinding was not needed.

Reporting for specific materials, systems and methods

We require information from authors about some types of materials, experimental systems and methods used in many studies. Here, indicate whether each material, system or method listed is relevant to your study. If you are not sure if a list item applies to your research, read the appropriate section before selecting a response.

Materials & experimental systems

n/a	Involved in the study
☐	☒ Antibodies
☒	☐ Eukaryotic cell lines
☒	☐ Palaeontology and archaeology
☒	☐ Animals and other organisms
☐	☒ Clinical data
☒	☐ Dual use research of concern

Methods

n/a	Involved in the study
☒	☐ ChIP-seq
☒	☐ Flow cytometry
☐	☒ MRI-based neuroimaging

Antibodies

Antibodies used	CD3 (mouse clone LN10) IgG1 antibody (#NCL-L-CD3-565) was purchased from Leica Biosystems (Wetzlar, Germany). The CD8 (mouse clone C8/144B) IgG1κ antibody (#M7103) and the Ki-67 (mouse clone MIB-1) IgG1κ antibody (#M7240) were purchased from Dako (Santa Clara, CA). Antibodies were diluted per the manufacturer's instructions.
Validation	Data and references for the validation of the anti-human antibodies for the applications described here are provided by the suppliers (https://shop.leicabiosystems.com/en-gb/ihc-ish/ihc-primary-antibodies/pid-cd3 and https://www.agilent.com/en/product/immunohistochemistry/antibodies-controls/primary-antibodies)

Clinical data

Policy information about clinical studies

All manuscripts should comply with the ICMJE guidelines for publication of clinical research and a completed CONSORT checklist must be included with all submissions.

Clinical trial registration	NCT03343197
Study protocol	The full study protocol and statistical analysis plan will be provided with the manuscript.
Data collection	Data were collected at seven clinical sites in the United States: the Memorial Sloan Kettering Cancer Center, University of California Los Angeles Medical Center, University of Texas Southwestern, University of California San Francisco Division of Neuro-Oncology, Dana Farber Cancer Institute, Massachusetts General Hospital, and Duke University Medical Center.
Outcomes	The primary end point of 2-HG concentration in resected tumors was evaluated by comparing concentrations in patients with mIDH1 glioma treated with vorasidenib or ivosidenib with concentrations in tumors from untreated on-study patients (internal contemporaneous control) and additional tumors from untreated patients with wild-type IDH (N =15) and mIDH1 (N = 61) glioma (external control using previously banked tumor samples). Concentration of 2-HG was measured in tumor and plasma using liquid chromatography with tandem mass spectrometry. The Bayesian hierarchical model was applied to analyze the 2-HG concentration on a log10 scale to compare the treated groups with the untreated group. The model can dynamically borrow 2-HG concentration information between banked untreated frozen reference samples and the enrolled untreated control subjects, which results in power gain where two data sources (banked frozen reference samples and untreated subjects in this study) are similar, while controlling bias in cases where the two data sources differ dramatically. The posterior mean and 95% credible interval of 2-HG concentrations for each treatment group and the difference between each treated group and the untreated group was provided. Secondary objectives were to evaluate the safety profile of ivosidenib and vorasidenib, to evaluate the changes in 2-HG concentration in plasma pre- and post-treatment compared with untreated controls, to evaluate the pharmacokinetics of ivosidenib or vorasidenib in tumor tissue and plasma, and to evaluate the preliminary clinical activity of ivosidenib or vorasidenib monotherapy in the residual disease setting as assessed by modified Response Assessment in Neuro-oncology (RANO) for LGG.

Magnetic resonance imaging

Experimental design

Design type

We used a standardized acquisition for MRIs, and the Screening eligibility was centralized. The study manual specified: MRI Brain examinations should adhere to the International Standardized Brain Tumor Imaging Protocol for Multicenter Clinical Trials (Ellingson BM, et al. Consensus recommendations for a standardized brain tumor imaging protocol in clinical trials. Neuro Oncol 2015; 17(9): 1188-98), with the addition of 3D T2-weighted FLAIR images, when possible, in order to maximize detection, measurement and evaluation of tumor response to therapy. The study manual included MRI Brain guidelines for acquisition. All clinical sites were qualified by the vendor and required to perform the mandatory sequences at each time point. In addition, sites were instructed to use the same method of assessment, same imaging techniques, and the same equipment/scanners whenever possible to ensure consistency.

Design specifications

Specify the number of blocks, trials or experimental units per session and/or subject, and specify the length of each trial or block (if trials are blocked) and interval between trials.

Behavioral performance measures

State number and/or type of variables recorded (e.g. correct button press, response time) and what statistics were used to establish that the subjects were performing the task as expected (e.g. mean, range, and/or standard deviation across subjects).

Acquisition

Imaging type(s)

Specify: functional, structural, diffusion, perfusion.

Field strength

Specify in Tesla

Sequence & imaging parameters

Specify the pulse sequence type (gradient echo, spin echo, etc.), imaging type (EPI, spiral, etc.), field of view, matrix size, slice thickness, orientation and TE/TR/flip angle.

Area of acquisition

State whether a whole brain scan was used OR define the area of acquisition, describing how the region was determined.

Diffusion MRI ☐ Used ☐ Not used

Preprocessing

Preprocessing software

Provide detail on software version and revision number and on specific parameters (model/functions, brain extraction, segmentation, smoothing kernel size, etc.).

Normalization

If data were normalized/standardized, describe the approach(es): specify linear or non-linear and define image types used for transformation OR indicate that data were not normalized and explain rationale for lack of normalization.

Normalization template

Describe the template used for normalization/transformation, specifying subject space or group standardized space (e.g. original Talairach, MNI305, ICBM152) OR indicate that the data were not normalized.

Noise and artifact removal

Describe your procedure(s) for artifact and structured noise removal, specifying motion parameters, tissue signals and physiological signals (heart rate, respiration).

Volume censoring

Define your software and/or method and criteria for volume censoring, and state the extent of such censoring.

Statistical modeling & inference

Model type and settings

Specify type (mass univariate, multivariate, RSA, predictive, etc.) and describe essential details of the model at the first and second levels (e.g. fixed, random or mixed effects; drift or auto-correlation).

Effect(s) tested

Define precise effect in terms of the task or stimulus conditions instead of psychological concepts and indicate whether ANOVA or factorial designs were used.

Specify type of analysis: ☐ Whole brain ☐ ROI-based ☐ Both

Statistic type for inference
(See Eklund et al. 2016)

Specify voxel-wise or cluster-wise and report all relevant parameters for cluster-wise methods.

Correction

Describe the type of correction and how it is obtained for multiple comparisons (e.g. FWE, FDR, permutation or Monte Carlo).

Models & analysis

n/a	Involved in the study
☐	☐ Functional and/or effective connectivity
☐	☐ Graph analysis
☐	☐ Multivariate modeling or predictive analysis

Functional and/or effective connectivity	*Report the measures of dependence used and the model details (e.g. Pearson correlation, partial correlation, mutual information).*
Graph analysis	*Report the dependent variable and connectivity measure, specifying weighted graph or binarized graph, subject- or group-level, and the global and/or node summaries used (e.g. clustering coefficient, efficiency, etc.).*
Multivariate modeling and predictive analysis	*Specify independent variables, features extraction and dimension reduction, model, training and evaluation metrics.*
